# MYDGF as a telomerase activator and therapeutic target for osteoarthritis

**DOI:** 10.1007/s44307-026-00119-6

**Published:** 2026-08-31

**Authors:** Yijun He, Chujun Li, Kexin Chen, Xining Wang, Shengxin Xu, Zhifen Zhou, Li Yin, Jialu Zheng, Yifan Wu, Wenbin Ma, Zhou Songyang, Yan Huang

**Affiliations:** 1 MOE Key Laboratory of Gene Function and Regulation, State Key Laboratory of Biocontrol and Guangzhou Key Laboratory of Healthy Aging Research, School of Life Sciences, Sun Yat-Sen University, Guangzhou, 510275 China; 2 Hainan Academy of Medical Sciences, Hainan Medical University, Haikou, 571199 China

**Keywords:** Osteoarthritis (OA), Cartilage degeneration, Myeloid-derived growth factor (MYDGF), Telomerase

## Abstract

Osteoarthritis (OA) involves progressive cartilage breakdown. This is driven by impaired chondrocyte function and an imbalance in the extracellular matrix homeostasis. However, the key upstream regulators that maintain healthy chondrocytes are still not fully known. Here, we report that myeloid-derived growth factor (MYDGF) is a novel regulator of telomerase activity and is critical for cartilage health. Using a genome-wide CRISPR-Cas9 screen with a TERT reporter system, MYDGF was identified as a strong positive regulator of telomerase. Functional studies confirmed that MYDGF positively regulates TERT expression and telomerase activity in both HeLa and ATDC5 cells. In a mouse surgical destabilization of the medial meniscus (DMM) OA model, MYDGF knockout (KO) mice exhibited more severe cartilage damage. Conversely, delivering MYDGF into the knee joint using AAV virus partially alleviated cartilage injury. Transcriptome profiling revealed that MYDGF-deficient chondrocytes exhibit downregulated key pathways involved in matrix building and upregulated inflammatory signals, indicating a shift in cell state. In summary, our work establishes MYDGF as a key upstream factor for both telomerase and cartilage matrix homeostasis remodeling. It connects telomerase function to chondrocyte health. These findings also highlight MYDGF as a promising new target for treating osteoarthritis.

**Supplementary Information:**

The online version contains supplementary material available at https://doi.org/10.1007/s44307-026-00119-6.

## Introduction

Aging is an inevitable biological process characterized by progressive deterioration of structure and function, and it has evolved from an individual physiological concern into a major societal challenge, thereby making the elucidation of its cellular and molecular mechanisms and the development of strategies to delay aging, a critical task in modern biomedicine.

From a macroscopic perspective, organismal aging is characterized by fourteen major hallmarks (López-Otín and Blasco et al., 2013; López-Otín and Blasco et al., 2023; Kroemer and Maier et al., 2025). Among these, telomere attrition serves as a critical link between cellular senescence and organismal aging and has been validated across multiple animal models. Mice possessing exceptionally long telomeres exhibit extended lifespan (Mu Oz-Lorente and Cano-Martin et al., 2019), whereas mice with shortened telomeres display markedly reduced lifespan phenotypes (Armanios and Alder et al., 2009).

To prevent the onset of replicative senescence and maintain genomic homeostasis, telomeres preserve their length and stability through specialized elongation mechanisms: telomerase-dependent telomere elongation and telomerase-independent alternative lengthening of telomeres (ALT). The majority of telomere-elongating cells rely predominantly on telomerase activity for telomere maintenance, whereas only a small subset of tumor cells employ the ALT pathway to extend telomeres (Maciejowski and de Lange 2019). In humans, most normal somatic cells lack telomerase activity. With increasing age, telomeres are progressively eroded, driving cells and tissues into a state of growth arrest and ultimately contributing to organismal aging. Common age-related diseases, such as chronic obstructive pulmonary disease, osteoporosis, and chronic lung disorders, have also been shown to be closely associated with telomere shortening (Rossiello and Jurk et al., 2022). Accordingly, the regulation of telomere length by telomerase activity is under strict surveillance and limitation in the human body, with telomerase activity is typically confined to a small subset of cells, including stem cells, cancer cells, and certain specialized somatic cells (Shay and Wright 2019).

Chondrocyte senescence serves as a crucial cellular basis for the onset and progression of osteoarthritis, characterized by diminished replicative capacity and accompanied by mitochondrial dysfunction, epigenetic abnormalities, and the activation of classic cell cycle inhibitory pathways (p53/p16/p21). These factors collectively contribute to the disruption of cartilage homeostasis and the advancement of the disease. Specifically, the senescence of human articular chondrocytes is closely associated with telomere shortening. Studies have shown that with increasing age, senescence-associated β-galactosidase activity rises, while both cell proliferative capacity and telomere length significantly decline, indicating that telomere shortening in chondrocytes is an important marker of age-related senescence. Under conditions of long-term injury and chronic stress, persistent telomere shortening can accelerate the process of chondrocyte senescence, thereby promoting the development and progression of osteoarthritis (Martin and Buckwalter 2002). Furthermore, oxidative stress and impaired mitochondrial respiratory chain function lead to ROS accumulation, aberrant DNA methylation and histone modifications that regulate the expression of OA-related genes (such as OP-1, MMPs, and ADAMTS) and activate the p53-p21 and p16-Rb pathways which mediate cell cycle arrest. These are significant mechanisms driving chondrocyte senescence and OA progression (Roach and Yamada et al., 2005; Passos and Nelson et al., 2010; Childs and Durik et al., 2015).

Existing evidence indicates that osteoarthritis leads to progressive degeneration and functional impairment of the entire joint, accompanied by chondrocyte loss, inflammatory responses, and dysregulation of extracellular matrix (ECM) homeostasis. Complex signaling pathways determine cell fate, among which the PI3K/Akt/mTOR signaling pathway is essential for maintaining joint health and is implicated in the pathogenesis of osteoarthritis. The PI3K/AKT pathway contributes to ECM synthesis by upregulating the expression of Col2a1 and aggrecan. Additionally, it exerts a protective effect against cartilage injury in rats through the downregulation of Mmp13 expression (Iwasa and Hayashi et al., 2014; Zhang and Lai et al., 2018). At the chondrocyte level, the activation or inhibition of Akt determines whether cells undergo proliferation or apoptosis (Stanic and Facchini et al., 2006). These processes exert an influence on the homeostatic remodeling of the cartilage matrix.

MYDGF (myeloid-derived growth factor) is a multifunctional secretory protein widely involved in various physiological and pathological processes. It was initially identified in 2001 and has also been referred to as stromal cell-derived growth factor (SF20) or interleukin-25 (IL-25) (Tulin and Onoda et al., 2003). MYDGF is expressed in a variety of cell types, including hepatocellular carcinoma cells (Sunagozaka and Honda et al., 2011), adipocytes (Wang and Mariman et al., 2004), human synovial cells (Weiler and Bellmann 2007), and bone marrow-derived macrophages, and has been shown to play important roles in cardiac repair (Korf-Klingebiel and Reboll et al., 2015), diabetic nephropathy (He and Li et al., 2020), hepatocellular carcinoma development (Wang and Li et al., 2020), and inflammatory responses (Meng and Li et al., 2021). Structurally, human MYDGF consists of 173 amino acids, and nuclear magnetic resonance (NMR) analysis reveals that it is composed of α-helices and β-sheets, with its potential receptor-interacting interface preliminarily predicted (Bortnov and Tonelli et al., 2019).

The definitive function of this protein was elucidated through research on acute myocardial infarction. Korf-Klingebiel et al. discovered that bone marrow-derived cells secrete MYDGF to promote cardiomyocyte survival and angiogenesis, playing a critical role in cardiac injury repair. Its molecular mechanism may involve the activation of classic cell survival signaling pathways such as MAPK/Akt (Korf-Klingebiel and Reboll et al., 2015). Beyond the cardiovascular field, MYDGF also demonstrates therapeutic potential in other disease models. In diabetic nephropathy, MYDGF expression is downregulated, and supplementation with MYDGF may protect renal function by modulating autophagy and inhibiting podocyte apoptosis. Recent studies have further shown that MYDGF expression is associated with the efficacy of anti-fibrotic drugs, suggesting its potential role in suppressing organ fibrosis (Wilson and Chiles et al., 2022). To address potential issues such as short half-life when used as a therapeutic protein, researchers have employed protein engineering strategies by fusing MYDGF with a specific region of human CD164, successfully creating a novel fusion protein MYDGF164 with significantly prolonged half-life. This fusion protein retains its original biological activity and demonstrates efficacy in alleviating renal fibrosis in chronic kidney disease models. These studies provide a crucial theoretical foundation for the future development of MYDGF as a biological therapeutic for myocardial infarction, fibrotic diseases, and potentially degenerative joint disorders.

Of particular interest is its potential association with aging-related diseases such as osteoarthritis. Aging is a major risk factor for osteoarthritis, accompanied by the senescence of articular chondrocytes, one of the underlying mechanisms of which is telomere shortening (telomere senescence). Studies have found that telomere length is shorter in cells derived from osteoarthritic lesion compared to cells in distal areas from the lesion, indicating that telomere dysfunction and cellular senescence play key roles in disease progression (Price and Waters et al., 2002). Previous studies have shown that the expression of the MYDGF protein can be detected in the synovial tissue of the bone joints. This suggests that MYDGF may act as an endogenous protective factor within the joint microenvironment.

## Methods

### Mice

All experimental animals used were 8-week-old male C57BL/6 J mice, weighing 20–30 g. The wild-type (WT) and MYDGF knockout (KO) mice employed in this study were obtained by intercrossing MYDGF heterozygous mice, which were kindly provided by Prof. Yu Nie from the Chinese Academy of Medical Sciences (Peking Union Medical College Hospital). The mice were housed under specific pathogen-free (SPF) conditions with a 12-h light/dark cycle, at a controlled temperature of 22 ± 2 °C and humidity of 50–60%. The animals had ad libitum access to food and water and were allowed free movement within their cages. All animal experiments were performed in accordance with the National Institutes of Health Guide for the Care and Use of Laboratory Animals and the ARRIVE guidelines.

### Cell culture

Human cervical cancer HeLa cells (HeLa, RRID: CVCL_0030), human embryonic kidney 293 T cells (HEK 293 T, RRID: CVCL_0063), mouse embryonic stem cells ES-E14TG2a cells (E14, RRID: CVCL_9108), human monocytic U937 cells (U937, RRID: CVCL_0007), human promyelocytic leukemia HL60 cells (HL60, RRID: CVCL_0002), human prostate cancer PC3 cells (PC3, RRID: CVCL_0035), human prostate cancer DU145 cells (DU145, RRID: CVCL_0105), and human near-haploid HAP1 cells (HAP1, RRID: CVCL_Y019) were obtained from the American Type Culture Collection (ATCC, Manassas, VA, USA). Mouse chondrogenic ATDC5 cells (ATDC5, RRID: CVCL_3894) were kindly provided by Prof. Xiaochun Bai from Southern Medical University.

Primary chondrocytes were isolated from femoral heads of 5-day-old C57 neonatal mice. Tissues were digested overnight with 0.2% type II collagenase (Gibco, Cat. No. 17101015) at 37℃, centrifuged, and resuspended in DMEM/F-12 (Gibco, Cat. No. C11330500BT) with 10% fetal bovine serum (Hyclone Cat. No. SH30084.03) and 1% penicillin-streptomycin (PS, NCMbio, C100C5) for culture at 37℃ in a humidified incubator with 5% CO2. Cells were passaged at 90–95% confluency. After removing the supernatant and washing with PBS, cells were digested with 0.25% trypsin-EDTA (T25, Gibco, 25200072) at 37 °C for 1 min until detachment. Digestion was terminated by adding complete medium with 10% serum, followed by gentle pipetting to collect the cell suspension. After centrifugation at 1200 g for 3 min, the pellet was resuspended in fresh complete medium and reseeded at a 1:2 or 1:3 split ratio into new dishes. Cell medium was changed every 2–3 days and cells were used at passage 2–4.

HeLa, 293 T, PC3, DU145, HAP1 and ATDC5 cells were cultured in DMEM (Gibco, Cat. No. C11885500BT) supplemented with 10% fetal bovine serum (FBS, ExCell Bio, FSP500) and 1% PS (NCMbio, C100C5) at 37℃ in a humidified incubator with 5% CO2 and passaged as primary chondrocytes. U937 and HL60 cells were cultured in Roswell Park Memorial Institute (RPMI) 1640 (Gibco, Cat. No. 11875093) supplemented with 10% fetal bovine serum (FBS, ExCell Bio, FSP500) and 1% PS (NCMbio, C100C5) at 37℃ in a humidified incubator with 5% CO2, and passaged by centrifugation and resuspension in fresh medium. Mouse E14 cells were cultured in DMEM (Gibco, Cat. No. C11885500BT) supplemented with 15% fetal bovine serum (Hyclone, Cat. No. SH30084.03), 1× GlutaMAX (Gibco, Cat. No. 35050061), 1× non-essential amino acids (Gibco, Cat. No. 11140050), 1% PS (NCMbio, Cat. No. C100C5), 0.1 mM 2-mercaptoethanol (Sigma-Aldrich, Cat. No. M3148), 1 μM PD0325901 (Cayman, Cat. No. 13034), 3 μM CHIR99021 (Cayman, Cat. No. 13122), and 1000 U/mL leukemia inhibitory factor (LIF; Gibco, Cat. No. A35933). Cells were maintained at 37 °C in a humidified incubator with 5% CO₂. To test for mycoplasma contamination, 100 μL of cell culture supernatant was collected and subjected to PCR using specific primers targeting the mycoplasma 16S rRNA gene. The PCR reaction mixture contained 2 × Rapid Taq Master Mix (Vazyme, P222), forward and reverse primers. Thermal cycling was performed with an initial denaturation at 95 °C for 5 min, followed by 28 cycles of 95 °C for 15 s, 56 °C for 15 s, and 72 °C for 15 s, with a final extension at 72 °C for 5 min. Both positive and negative controls were included in each run. The PCR products were analyzed by electrophoresis on a 1–2% agarose gel, where a band of approximately 200–300 bp indicated positive contamination. All cell lines used in this study were routinely tested and confirmed negative for mycoplasma. The primer sequence information is as follows:

Myco-FP, GGGAGCAAACAGGATTAGTATCCCT;

Myco-RP, TGCACCATCTGTCACTCTGTTAACCTC.

### Stable cell transfection

For lentivirus production, HEK293T cells at 40–50% confluence were transfected using plasmid DNA (transfer plasmid:psPAX2:pMD2.G = 4:3:1) and PEI (1:3.3 mass ratio) in Opti-MEM. After 15 min incubation, the complex was added to cells. Fresh medium was replaced 6–8 h later, and viral supernatant was collected at 48 and 72 h, filtered with 0.45 μm membrane filter (JET BIOFIL, China), and used to infect target cells with 10 μg/mL Polybrene (Sigma-Aldrich, USA). Medium was changed after 6–8 h. Antibiotic selection started 48 h post-infection until all non-infected control cells died. The shRNA/knockdown-related sequence information is as follows:

**Table:** 

Gene name	shRNA target sequence(5' → 3')
mMydgf	GCACTTTACCTGTACCATC
	GCTGAGATCGAGTATGCCA

### sgRNA design and transfection

Single-guide RNAs (sgRNAs) targeting the gene of interest were designed using an online CRISPR design tool and synthesized accordingly. The sgRNA sequences were cloned into the appropriate CRISPR/Cas9 expression vectors. Cells were transfected with sgRNA constructs together with Cas9 expression plasmids using standard transfection reagents according to the manufacturer’s instructions. After transfection, cells were cultured under appropriate conditions for subsequent analyses. The sequences of sgRNAs used in this study are listed below.

**Table:** 

Gene	Number	Forward Primer(5’ → 3’)	Reverse Primer(5’ → 3’)
ASXL2	−1	CACCGTGGGCGGAGGCCGCCAAGA	AAACTCTTGGCGGCCTCCGCCCAC
	−2	CACCGTTATGACTCATGGGTGTAT	AAACATACACCCATGAGTCATAAC
CTNND2	−1	CACCGCGGCTTAAACACCTCCAAC	AAACGTTGGAGGTGTTTAAGCCGC
	−2	CACCGTGTTTGCGAGGAAGCCGCC	AAACGGCGGCTTCCTCGCAAACAC
C1orf210	−1	CACCGGGCATGGTATCGGCGGCAG	AAACCTGCCGCCGATACCATGCCC
	−2	CACCGCCGGTAGCTGGCATGGTAT	AAACATACCATGCCAGCTACCGGC
MYDGF	−1	CACCGTGTGGGCCGCGCTGCTCCTA	AAACTAGGAGCAGCGCGGCCCACAC
	−2	CACCGCAAGATACTCACGTAGGCCA	AAACTGGCCTACGTGAGTATCTTGC
	−3	CACCGCGCTGAGATTGAGTACGCCA	AAACTGGCGTACTCAATCTCAGCGC

### Isolation and culture of primary mouse chondrocytes

#### Western blot

Cells were lysed with RIPA buffer containing protease and phosphatase inhibitors (Protease Inhibitor Cocktail: Selleck, B14001; Phosphatase Inhibitor: Servicebio, G2007) on ice for 30 min, followed by sonication and centrifuged at 4 ℃ (14,000 g, 15 min). The supernatants were collected, protein concentrations were measured using a BCA assay and the samples were denatured in loading buffer by boiling for 10 min. Equal amounts (20 μg) for total protein were loaded per lane. Proteins were separated by SDS-PAGE (80 V for 20 min, then 150 V) and transferred to NC membrane via semi-dry transfer. After blocking with 5% skim milk for 1 h at room temperature, the membrane was incubated with primary antibody (2 h at RT or overnight at 4 ℃), washed, incubated with secondary antibody (1 h at RT), washed again, and finally visualized using an Odyssey Infrared Imaging System (LI-COR Biosciences, Model 9120).

**Table:** 

Antibody	Catalog
mouse anti-Flag Tag (1:1000)	F1804, Sigma-Aldrich, USA
mouse anti-His Tag (1:5000)	A00186, GenScript, China
mouse anti-GAPDH (1:5000)	M20006, Abmart, China
mouse anti-Tubulin (1:5000)	T6074, Sigma-Aldrich, USA
goat anti-SF20/MYDGF (1:1000)	AF1147, R&D Systems, USA
mouse anti-Akt (pan) (1:1000)	2920, Cell Signaling Technology, USA
rabbit anti-Phospho-Akt(Ser473) (1:1000)	9271, Cell Signaling Technology, USA
IRDye 800CW Goat anti-Rabbit IgG (H + L)IRDye 800CW Goat anti-Rabbit IgG (H + L) (1:1000)	926–32,211, LI-COR, USA
IRDye 680RD Goat anti-Mouse IgG (H + L)IRDye 680RD Goat anti-Mouse IgG (H + L) (1:1000)	926–68,070, LI-COR, USA

### RNA extraction and reverse transcription

Cells were lysed with Trizol reagent (Invitrogen, 15596018CN) after PBS washing. Following chloroform addition and centrifugation, the aqueous phase was mixed with isopropanol to precipitate RNA. The pellet was washed twice with 75% ethanol, air-dried, and dissolved in RNA-free water. RNA concentration was measured by NanoDrop 2000 spectrophotometer (Thermo Scientific, USA), and 1 μg RNA was used for reverse transcription according to the PrimeScript RT reagent kit (Takara, RR037A).

### Real-time fluorescence quantitative PCR

The real-time fluorescence quantitative PCR was performed using a reaction mixture consisting of 5 µL of 2 × TB Green PCR Master Mix (Takara, RR820A), 0.2 µL each of 10 µM forward and reverse primers, 1 µL of template cDNA, and ddH_2_O added to bring the total volume to 10 µL. The thermal cycling protocol included an initial pre-denaturation step at 95 °C for 5 min, followed by 40 cycles of denaturation at 95 °C for 5 s and annealing/extension at 60 °C for 1 min. The real-time fluorescence quantitative PCR assay was conducted on a qTOWER3 instrument (Analytik Jena, Germany), and the results were analyzed and expressed using the 2^−ΔΔCt^ method.

**Table:** 

Gene	Forward Primer (5' → 3')	Reverse Primer (5' → 3')
mMydgf	AGCGGAGGCTTCTGGACT	GTGCTGGCTGTCTTCACTTG
mCol2a1	GGGTCACAGAGGTTACCCAG	ACCAGGGGAACCACTCTCAC
mCol10a1	TTCTGCTGCTAATGTTCTTGACC	GGGATGAAGTATTGTGTCTTGGG
mAggrecan	CATCACAGAGTCCGAGTGGG	CACTCGTCAATGTCTGCAACG
mMmp13	TGTTTGCAGAGCACTACTTGAA	CAGTCACCTCTAAGCCAAAGAAA
mP16	CGCAGGTTCTTGGTCACTGT	TGTTCACGAAAGCCAGAGCG
mBcl2	GCTACCGTCGTGACTTCGC	CCCCACCGAACTCAAAGAAGG
mAdamts55	GCTCATGAAATTGGGCATCT	TCTGCTTCCGTGGTAGGTCT
mTubulin	AAGCAGCAACCATGCGTGA	CCTCCCCCAATGGTCTTGTC
hTERT	TCACGGAGACCACGTTTCAAA	TTCAAGTGCTGTCTGATTCCAAT
hMYDGF	CGAAGACCACCAGCACTTCA	GGCCATGGCGTACTCAATCT
hTubulin	AAGATCCGAGAAGAATACCCTGA	CTACCAACTGATGGACGGAGA

### Telomerase activity assay (Q-TRAP)

Cells were collected, washed with cold PBS, and lysed in cold NP-40 lysis buffer (10 mM Tris–HCl, pH 8.0, 150 mM NaCl, 1 mM MgCl_2_, 1 mM EDTA, 1% NP-40, 0.25% sodium deoxycholate, 10% glycerol) on ice for 30 min. After centrifugation at 4 ℃, the supernatant was collected. A standard curve was prepared using telomerase-positive samples. The reaction mixture (containing TS primer: 5’-AATCCGTCGAGCAGAGTT-3’, ACX primer: 5’-GCGCGGCTTACCCTTACCCTTACCCTAACC-3’, 10 mM EGTA, 2 × TB Green PCR Master Mix, and RNA-free water) was combined with cell lysate, incubated at 30 ℃ for 30 min in the dark for telomerase extension, then aliquoted into a 96-well plate (MIKXLIFE, China). Following centrifugation, Ct values were measured, and relative telomerase activity was normalized to total protein determined by BCA assay (Beyotime, P0012).

### CCK8

Cells were digested, counted, and seeded into 96-well plates at 1,000 cells per well (100 μL/well), with experimental, control, and blank (medium only) groups, each in quadruplicate. After 24 h, CCK-8 reagent (SIMUBIOTECH, SCK005) was added at 1/10 volume per well, gently mixed, and incubated at 37 ℃ for 2 h until color development. Absorbance was measured at 450 nm using a microplate reader (BioTek, Synergy H1).

### Genotyping

Mouse tail DNA was extracted using the KAPA kit (75 ℃ lysis, 100 ℃ inactivation). PCR was performed with primers P1-P3 and KAPA2G Mix under: 95 ℃ 3 min; 35 cycles of 95 ℃ 15 s, 55 ℃ 15 s, 72 ℃ 30 s; 72 ℃ 5 min. Genotyping was done by 2% agarose gel electrophoresis: WT 87 bp, Flox 185 bp, KO 248 bp.

### Modeling of medial meniscus instability

The medial meniscus destabilization (DMM) model induces osteoarthritis by transecting the medial meniscotibial ligament to cause joint instability and cartilage wear. Under anesthesia, a medial knee incision is made to cut the ligament, followed by suturing after confirming meniscal mobility. Postoperative care includes antibiotics and normal feeding.

### Construction of AAV virus

AAV was produced by transfecting 293 T cells at 60–70% confluency with a plasmid mixture (target:pRepcap:pHelper = 1:1:2) using PEI, followed by 96 h culture. Virus was harvested from supernatant and cells via PEG precipitation and sonication, then purified by iodixanol density gradient centrifugation, and concentrated by ultrafiltration with buffer exchange.

### Intra-articular injection

Four weeks post-DMM modeling, the anesthetized mouse’s right hind limb was disinfected, and 10 μL of AAV (1 × 10^11^ viral particles, determined by qPCR) was injected into the joint cavity via an insulin syringe inserted horizontally about 3 mm below the patella. The site was pressed and disinfected after injection.

### Collection, fixation and decalcification of mouse knee joint specimens

Mouse knee joints were harvested, washed with PBS, and fixed in 4% PFA at 4 °C for 48 h. After washing, tissues were decalcified in 10 volumes of 10% EDTA on a shaker at room temperature for approximately one month, with weekly solution changes.

### Paraffin embedding and sectioning of mouse knee joint specimens

Knee joints were washed with PBS, dehydrated through an ethanol series, cleared with TO clearing agent, and infiltrated with paraffin. Tissues were embedded sagittally, sectioned at 5 μm, floated on a 37 ℃ water bath, mounted on slides, and dried for storage.

### Mouse knee joint sections were stained with safranin O-solid green

Sections were deparaffinized in xylene and rehydrated through graded ethanol. After staining with Weigert's iron hematoxylin for 5 min and washing with water, sections were incubated in 0.1% Safranin O (pH 4.0) for 5 min to label proteoglycans in cartilage. Following a brief rinse in 1% acetic acid, sections were counterstained with 0.02% Fast Green for 2 min to label bone and collagen. Differentiation was achieved by rapid dehydration through 95% and 100% ethanol. Sections were cleared in xylene and mounted with neutral balsam.

### Micro-CT joint imaging

Mouse knee joints fixed in 4% PFA were scanned using a Micro-CT (Bruker) system with the following parameters: slice thickness 12 μm, voltage 55 kV, current 114 mA, scan time ~10 min per sample. After 3D reconstruction, a region of interest extending 2 cm above and below the joint line was defined based on the posterior condylar line. Axial sections were analyzed to evaluate differences in the subchondral bone plate and trabecular microstructure.

### Protein purification of prokaryotes

The prokaryotic expression plasmid was transformed into BL21 (DE3) cells, and a single colony was expanded. Protein expression was induced with 0.1 mM IPTG at 16 °C overnight when A600 reached 0.4–0.6. After sonication and centrifugation, the supernatant was purified by Ni–NTA affinity chromatography through equilibration, binding, washing, and elution steps. The column was regenerated with EDTA, NaOH, and ethanol for storage.

### Molecular cloning-related experiments

The molecular cloning procedure involves: PCR amplification using KOD FX (Toyobo, KFX-101) system (94 ℃ 2 min pre-denaturation, 98 ℃ 10 s denaturation, based on primer Tm ±5 ℃ annealing for 30 s, 68 ℃ extension at 1 kb/min for 25–40 cycles), followed by agarose gel electrophoresis verification; NotI/AscI (Thermo Fisher Scientific, KD0595/KD1894) double digestion of plasmids for validation; ligation of the target fragment into the pENTR vector using T4 DNA ligase (Vazyme, C301-01); LR (Thermo Fisher Scientific, 11791100) recombination to assemble the entry vector with the pLenti expression vector; and finally transformation of the recombinant plasmid into competent cells, plating, and extraction of monoclonal plasmids for downstream applications.

### Intracellular Ca^2+^ imaging

Rhod-2 AM (Yeasen, 40776ES) was dissolved in anhydrous DMSO to prepare a 5 mM stock solution, aliquoted, and stored at −20 °C protected from light. Before use, the stock solution was warmed to room temperature. Rhod-2 AM was diluted to 5 μM in HHBS buffer containing 0.04% Pluronic F-127 and 1 mM probenecid. Cells were washed three times with HHBS buffer, incubated with Rhod-2 AM working solution at 37 °C for 30 min, and washed three times with HHBS buffer. Subsequently, cells were stained with DAPI for 5 min at room temperature, and washed twice with HHBS buffer. Images were acquired using a fluorescence microscope.

### Statistical analysis

Data are expressed as mean ± SD and analyzed using GraphPad Prism (GraphPad Software, USA). Relative mRNA expression was calculated by the 2^⁻ΔΔCt^ method. Statistical significance was determined by two-tailed unpaired Student’s *t*-test for two-group comparisons or one-way ANOVA followed by Tukey’s post-hoc test for multiple-group comparisons. Significance levels are denoted as *p < 0.05, **p < 0.01, ***p < 0.001 and ****p<0.0001. Western blot bands were quantified using Image Studio Lite (LI-COR Biosciences, USA).

## Results

### Identification of MYDGF as a positive regulator of telomerase activity through a genome-wide CRISPR-Cas9 screen and functional validation

**Fig. 1:**
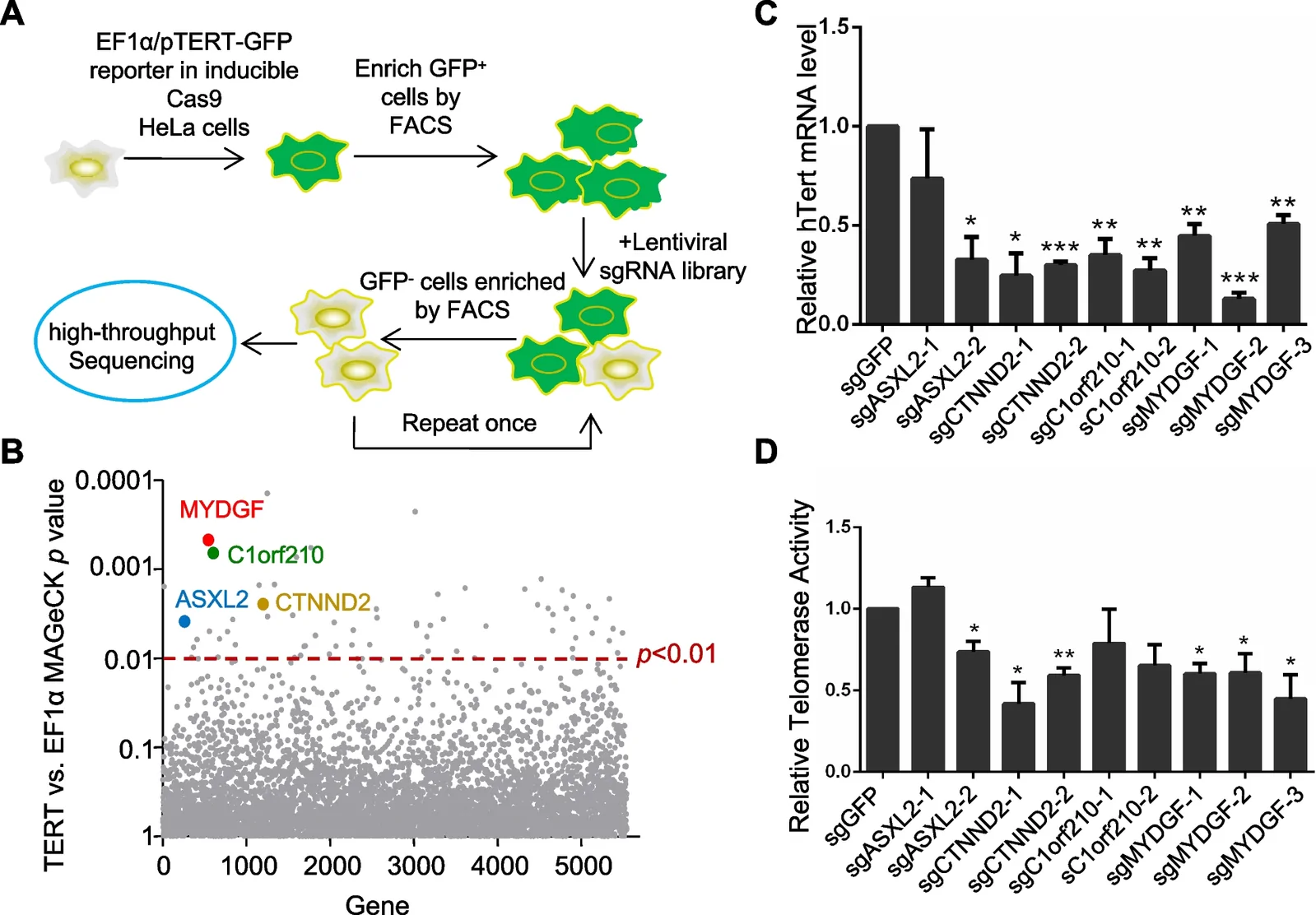
Identification and validation of MYDGF as a positive regulator of telomerase activity. **A** Schematic of a genome-wide CRISPR-Cas9 screen in HeLa cells carrying a pTERT-GFP reporter to identify regulators of TERT transcription. **B** MAGeCK analysis of enriched genes from GFP-negative populations. MYDGF was identified as a significant hit (*p* < 0.01). **C** Quantitative PCR analysis showing decreased hTERT mRNA levels normalized to hTubulin following MYDGF depletion in HeLa cells. **D** Quantification of telomerase activity in MYDGF-deficient HeLa cells. Statistical significance for panels C and D was determined by one-way ANOVA with Tukey's post hoc test. All data were presented as mean ± SD. Statistical significance was set at *p < 0.05, **p < 0.01, and ***p < 0.001. *n* = 3 biological replicates

In previous work conducted in our laboratory, a genome-wide CRISPR–Cas9 screen using a pTERT–GFP reporter in HeLa cells (Fig. 1A) identified DTX2 as a positive regulator of telomerase transcription (Zhou and Li et al., 2022). MAGeCK analysis revealed another four candidate genes, among which MYDGF was one of the most enriched genes in GFP-negative cells (Fig. 1B). CRISPR-based gene silencing was then used to reduce MYDGF expression. Knockdown of MYDGF led to decreased TERT mRNA levels (Fig. 1C), accompanied by a significant reduction in telomerase activity (Fig. 1D).

**Fig. 2:**
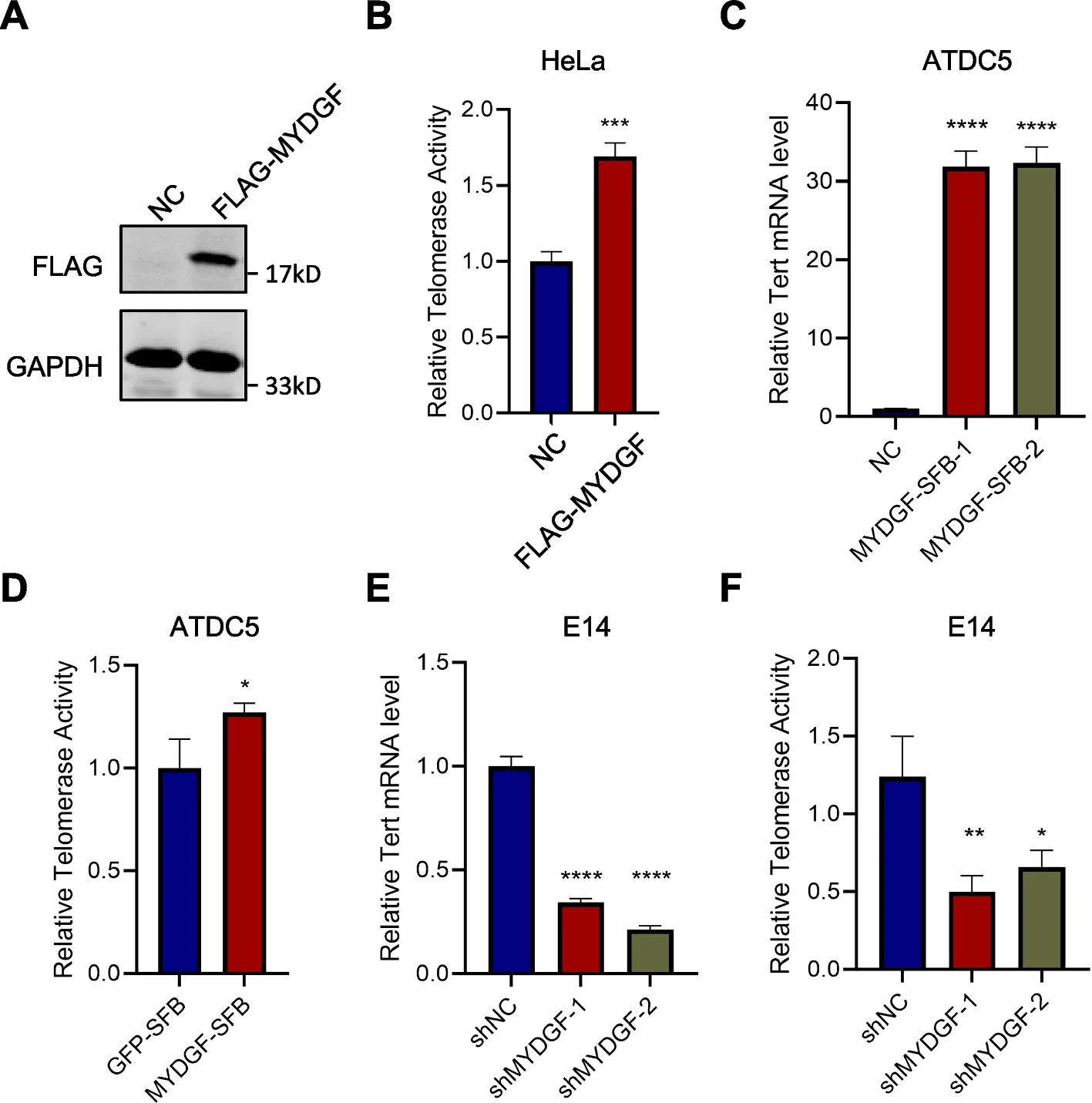
MYDGF positively regulates telomerase activity in multiple cell types. **A** Western-blot analysis showing MYDGF protein expression in HeLa cells transfected with empty vector negative control (NC) or Flag-tagged MYDGF. GAPDH was used as a loading control. **B** Relative telomerase activity in HeLa cells following MYDGF overexpression compared with negative control (NC). **C** Relative mRNA expression levels of MYDGF in ATDC5 cells transfected with negative control (NC), MYDGF-SFB-1, or MYDGF-SFB-2 plasmids, normalized to Tubulin. **D** Relative telomerase activity in ATDC5 cells transfected with GFP-SFB or MYDGF-SFB. **E **Relative MYDGF mRNA expression levels in E14 embryonic stem cells transfected with shNC, shMYDGF-1, or shMYDGF-2, normalized to Tubulin. **F** Relative telomerase activity in E14 cells transfected with shNC or shMYDGF-1/2. Statistical significance for panels B and D was determined using a two-tailed, unpaired Student's *t*-test, while panels C, E, and F were analyzed using one-way ANOVA with Tukey's post hoc test. All data were presented as mean ± SD. Statistical significance was set at *p < 0.05, **p < 0.01, ***p < 0.001 and ****p < 0.0001. *n* = 3 biological replicates

Similarly, expressing Flag-tagged MYDGF in HeLa cells increased telomerase activity compared to controls (Fig. 2A, B). In contrast, shRNA knockdown of MYDGF significantly lowered telomerase activity (Extended Data Fig. 1A-1B).

The same pattern occurred in ATDC5 cells. We first determined the characteristics of the ATDC5 cells (Extended Data Fig. 1C-1D). Overexpressing MYDGF there also raised telomerase activity (Fig. 2C, D). In ATDC5 cells, knocking down MYDGF with shRNAs effectively reduced its transcript levels. This led to a clear drop in telomerase activity (Extended Data Fig. 1E-1F). We also confirmed MYDGF is needed to maintain telomerase function in E14 embryonic stem cells (Fig. 2E, F).

Taken together, these results identify MYDGF as a positive regulator of telomerase activity. Both gain-of-function and loss-of-function studies consistently showed that MYDGF promotes TERT transcription and enhances telomerase activity. This effect was observed in HeLa cells, chondrocyte-lineage ATDC5 cells and E14 embryonic stem cells. Therefore, MYDGF is required to maintain telomerase function in a conserved role across different cell types.

### MYDGF activates telomerase through an endocytosis-dependent and calcium-mediated pathway

**Fig. 3:**
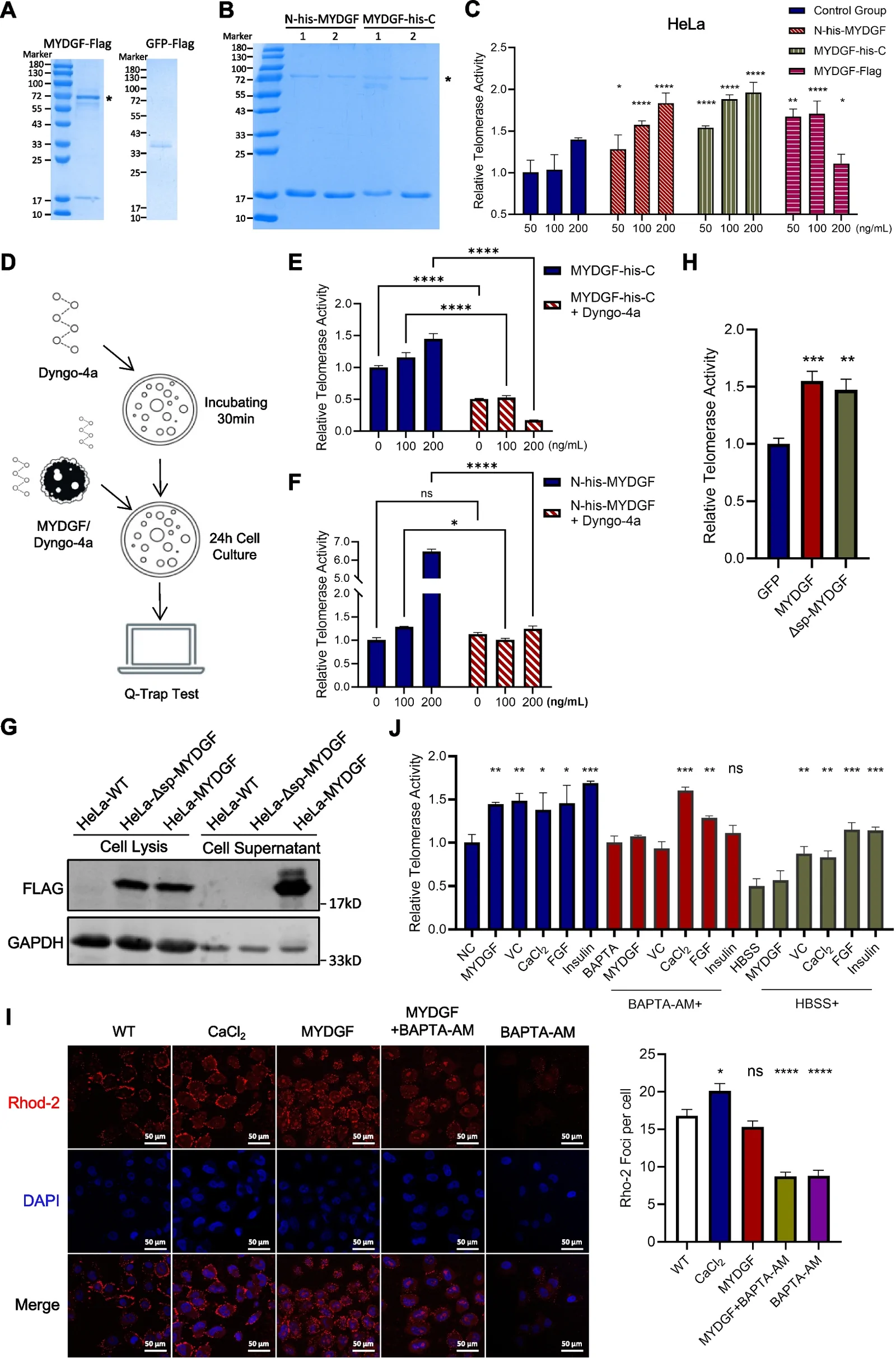
MYDGF protein enhances telomerase activity through calcium-dependent internalization and signaling. **A** Coomassie blue staining of recombinant MYDGF proteins purified from a eukaryotic expression system, MYDGF–Flag protein band is indicated by a red box. GFP–Flag protein was used as a control. Non-specific bands are indicated by asterisks (*). **B** Coomassie blue staining showing purified recombinant MYDGF protein expressed in *E. coli.*, including N-terminal His-tagged MYDGF (N-his-MYDGF) and C-terminal His-tagged MYDGF (MYDGF-his-C). Non-specific bands are indicated by asterisks (*). **C** Relative telomerase activity in HeLa cells treated with increasing concentrations of recombinant MYDGF proteins or control proteins, as measured by TRAP assay. **D** The experimental design for Dyngo-4a–mediated inhibition of MYDGF internalization. **E**, **F** Quantification of telomerase activity in HeLa cells treated with N-his-MYDGF (**E**) or MYDGF-his-C (**F**) in the presence or absence of Dyngo-4a, indicating that inhibition of endocytosis attenuates MYDGF-induced telomerase activation. **G** Western blot analysis of MYDGF protein levels in cell lysates and culture supernatants from HeLa cells expressing WT or ΔSP-MYDGF, confirming MYDGF secretion. **H** Relative telomerase activity in HeLa cells transfected with GFP, MYDGF overexpression plasmid, or ΔSP-MYDGF lacking the signal peptide. **I** Representative immunofluorescence images showing intracellular Ca^2+^ levels in HeLa cells stained with Rhod-2 following treatment with CaCl₂, MYDGF, MYDGF plus BAPTA-AM, or BAPTA-AM alone. Nuclei were counterstained with DAPI. Rhod-2 foci per cell were quantified. For each experiment, a minimum of 15 cells per condition were counted. **J** Relative telomerase activity in HeLa cells treated with MYDGF, Ca^2+^ modulators, or endocytosis inhibitors, indicating that MYDGF-induced telomerase activation depends on calcium signaling and cellular uptake. Statistical significance for panels C, E, F and J was determined using two-way ANOVA with Šídák's multiple comparisons test for comparisons between each treatment group and its corresponding control, while panels H and I were analyzed using one-way ANOVA with Tukey's post hoc test. All data were presented as mean ± SD. Statistical significance was set at *p < 0.05, **p < 0.01, ***p < 0.001 and ****p < 0.0001. *n* = 3 biological replicates

We successfully produced and purified recombinant MYDGF protein using both bacterial and mammalian systems. Analysis by Coomassie blue staining and immunoblot confirmed the protein's high purity and expected size (Fig. 3A, B). Adding purified MYDGF to cells increased telomerase activity in a dose-dependent manner. Control proteins like GFP did not produce this effect (Fig. 3C). We saw this stimulation in many different human cell lines, including U937, HL60, PC3, DU145, and HAP1 cells (Extended Data Fig. 2A-E). These experiments showed that externally applied MYDGF protein is sufficient to boost telomerase activity across different cell types.

Next, we wanted to understand how the protein works. We found that Dyngo-4a, an inhibitor of clathrin-mediated endocytosis, completely blocked MYDGF's ability to enhance telomerase (Fig. 3D-F). This means the protein must be taken up by cells to function. We also tested if the protein's own secretion pathway was important. Even when we disrupted general protein secretion by lacking the signal peptide, the added MYDGF protein could still increase telomerase activity (Fig. 3G, H). So, the active protein itself does not need to go through the secretory pathway to activate telomerase. Besides, Ca^2+^ is one of the most important second messengers in cells. Calcium homeostasis also plays a critical role in regulating telomerase through both transcriptional and post-translational mechanisms. Previous studies showed that elevated intracellular Ca^2+^ levels suppress telomerase in cell differentiation, through Ca^2+^-responsive signaling pathways and calcium-binding proteins that repress hTERT transcription (Rosenberger and Thorey et al., 2007). Ca^2+^ signaling influences telomerase activity by regulating the subcellular localization of TERT, as stress-induced Ca^2+^ elevation promotes nuclear export of TERT and reduces its telomere-elongating function (Diffendall and Dr. Resendes, 2015). Beyond the upstream regulation, accumulating evidence indicates that telomerase also exerts reciprocal control over cellular Ca^2+^ homeostasis. TERT has been shown to localize to mitochondria, where it modulates reactive oxygen species production, mitochondrial function, and intracellular Ca^2+^ balance through metabolic and stress-response pathways (Liu and Lin et al., 2024). Loss of TERT results in Ca^2+^ dysregulation and increased oxidative stress, suggesting that telomerase contributes to cellular homeostasis independently of telomere elongation (Miwa and Czapiewski et al., 2016). When we chelated intracellular calcium with 1,2-Bis(2-aminophenoxy)ethane-N,N,N',N'-tetraacetic acid tetrakis (acetoxymethyl ester) (BAPTA-AM), MYDGF could no longer activate telomerase (Fig. 3I, J). Depleting extracellular calcium with Hanks' Balanced Salt Solution (HBSS) also significantly reduced its effect (Fig. 3I, J). Therefore, MYDGF's function requires both intracellular and extracellular calcium.

In summary, our data support a model where externally added MYDGF protein first enters cells via endocytosis. Inside the cell, it then activates a calcium-dependent signaling pathway. This pathway ultimately leads to increased telomerase activity.

### MYDGF deficiency exacerbates cartilage degeneration in the DMM-induced osteoarthritis model

**Fig. 4:**
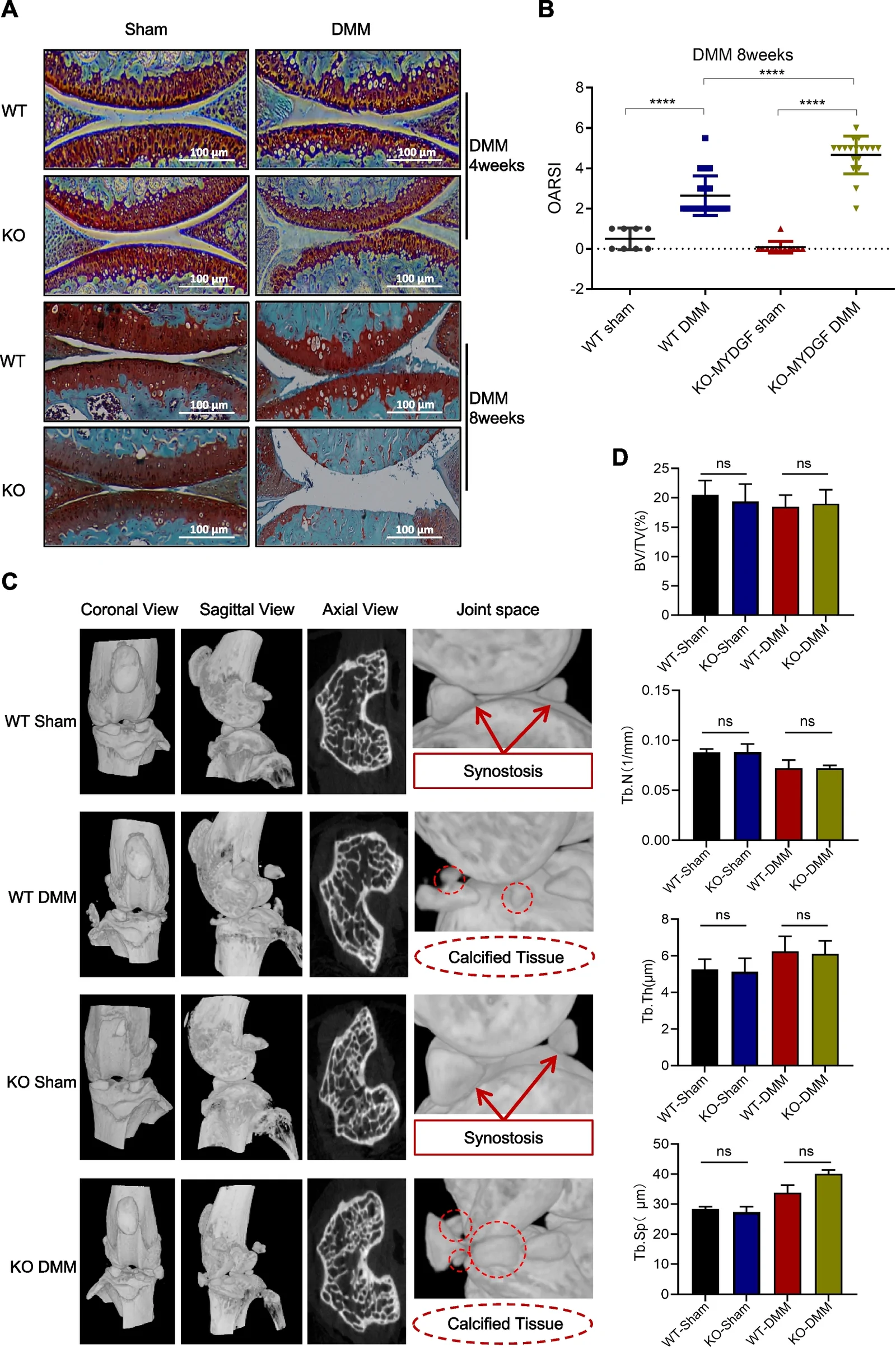
MYDGF deficiency exacerbates cartilage degeneration in the DMM-induced osteoarthritis model. **A** Representative Safranin O–Fast Green–stained sections of knee articular cartilage from WT and MYDGF KO mice subjected to sham operation or destabilization of the medial meniscus (DMM) surgery. Images were collected at 4 and 8 weeks after surgery. *n* = 22 mice. **B** Osteoarthritis Research Society International (OARSI) scores of articular cartilage from WT and MYDGF KO mice following sham or DMM surgery at 8 weeks post-operation. Each dot represents one mouse. Statistical significance was determined by one-way ANOVA with Tukey's post hoc test. *n* = 22 mice. **C** Representative micro-CT images of knee joints from WT and MYDGF KO mice subjected to sham or DMM surgery. Images are shown in coronal, sagittal, and axial planes, as well as three-dimensional reconstructions highlighting osteophyte formation and joint space alterations. *n* = 5 each group. **D** Quantitative micro-CT analysis of subchondral bone parameters, including bone volume fraction (BV/TV), trabecular number (Tb.N), trabecular thickness (Tb.Th), and trabecular separation (Tb.Sp), in WT and MYDGF KO mice following sham or DMM surgery. Statistical significance was determined by two-tailed unpaired Student's *t*-test. *n* = 5 each group. All data were presented as mean ± SD. Statistical significance was set at ****p < 0.0001, ns, not significant

Following DMM surgery, MYDGF KO mice developed more severe osteoarthritis than wild-type (WT) controls. Histological analysis showed exacerbated cartilage degradation and proteoglycan loss in MYDGF KO mice joints (Fig. 4A). This was confirmed by significantly higher OARSI scores at 8 weeks post-surgery, indicating a clear worsening of cartilage pathology (Fig. 4B).

Micro-computed tomography (CT) imaging revealed pronounced structural changes in KO mice (Fig. 4C), including increased osteophyte formation and joint space narrowing. Bone volume fraction (BV/TV) analysis shows that no significant change in bone mass and trabecular thickness (Tb.Th) between WT and KO mouse after DMM (Fig. 4D).

Together, these findings demonstrate that MYDGF deficiency accelerates osteoarthritis progression in vivo. Loss of MYDGF promotes both cartilage destruction and aberrant subchondral bone remodeling.

### Intra-articular delivery of AAV-MYDGF alleviates cartilage degeneration in the DMM-induced osteoarthritis model

**Fig. 5:**
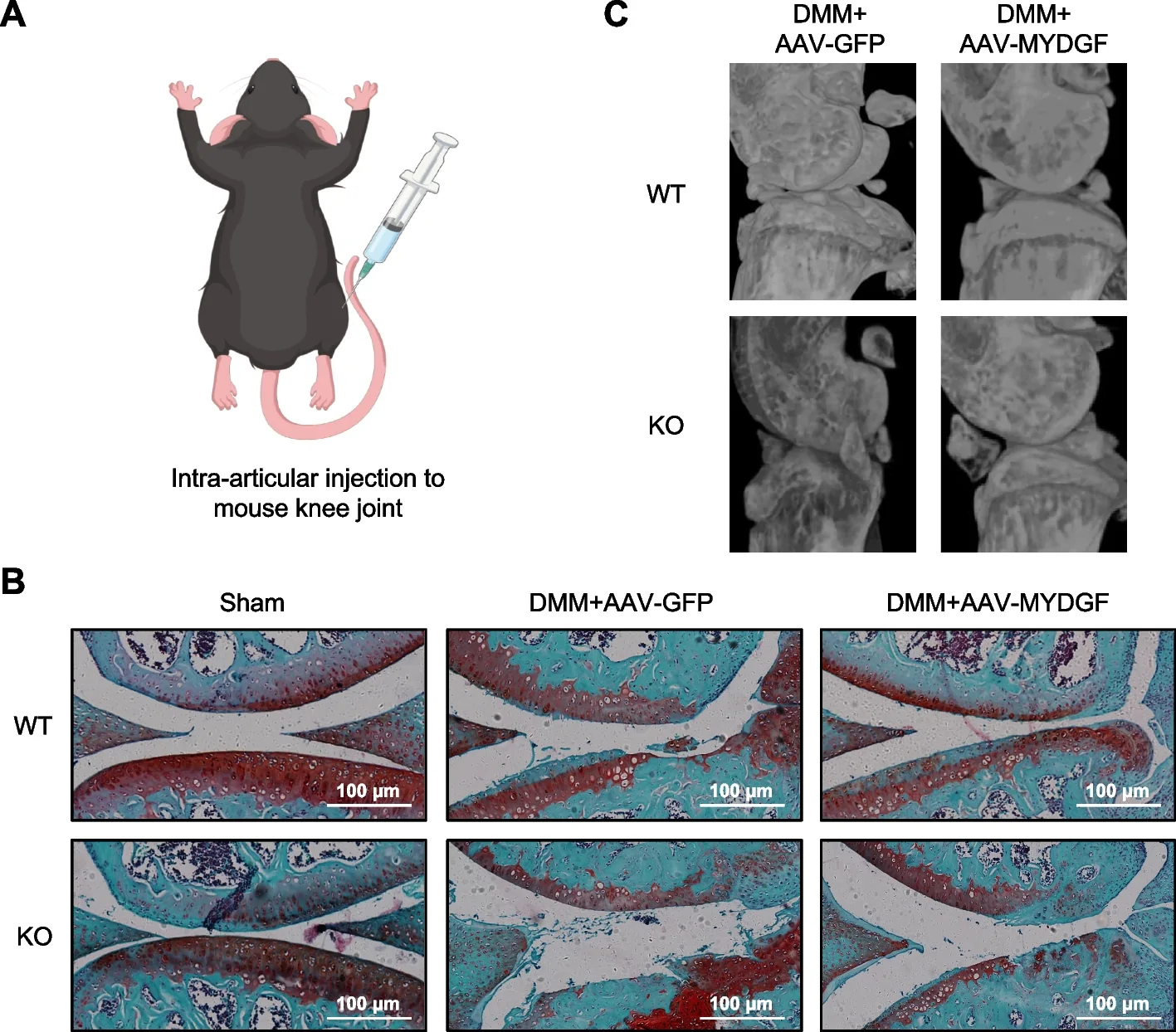
Intra-articular delivery of AAV-MYDGF alleviates cartilage degeneration in the DMM-induced osteoarthritis model. **A** Schematic illustration of intra-articular injection of adeno-associated virus (AAV) into the mouse knee joint. *n* = 3 each group. **B** Representative Safranin O–Fast Green–stained sections of knee articular cartilage from WT and MYDGF KO mice subjected to sham surgery or DMM surgery followed by intra-articular injection of AAV-GFP or AAV-MYDGF, as indicated. *n* = 3 each group. **C** Representative micro-CT images of knee joints from WT and MYDGF KO mice following DMM surgery and intra-articular injection of AAV-GFP or AAV-MYDGF. *n* = 3 each group

To evaluate the therapeutic potential of MYDGF, we performed intra-articular delivery of an adeno-associated virus expressing MYDGF (AAV-MYDGF) in mice subjected to DMM surgery. Control mice received AAV carrying GFP vector (AAV-GFP) (Fig. 5A). Expression of MYDGF was confirmed in 293 T cells (Extended Data Fig. 3).

Histological assessment was conducted 8 weeks post-surgery. Safranin O-Fast Green staining of knee joint sections revealed a clear protective effect (Fig. 5B). Cartilage from AAV-MYDGF-treated mice showed significantly less erosion and greater proteoglycan retention compared to the AAV-GFP group. The cartilage surface was also more intact. This confirms that elevating MYDGF levels in the joint mitigates injury-induced cartilage degeneration, supporting the therapeutic effect of MYDGF.

Finally, micro-CT analysis provided structural evidence (Fig. 5C). Joints treated with AAV-MYDGF displayed reduced osteophyte size and less subchondral bone sclerosis compared to the control group. This indicates that MYDGF overexpression moderates pathological bone remodeling.

In summary, local supplementation of MYDGF via AAV delivery effectively alleviates the progression of osteoarthritis in our model. It protects cartilage, reduces catabolic activity, and limits abnormal bone changes.

### MYDGF deficiency impairs chondrocyte proliferation and drives a catabolic transcriptomic shift via suppression of PI3K–Akt signaling

**Fig. 6:**
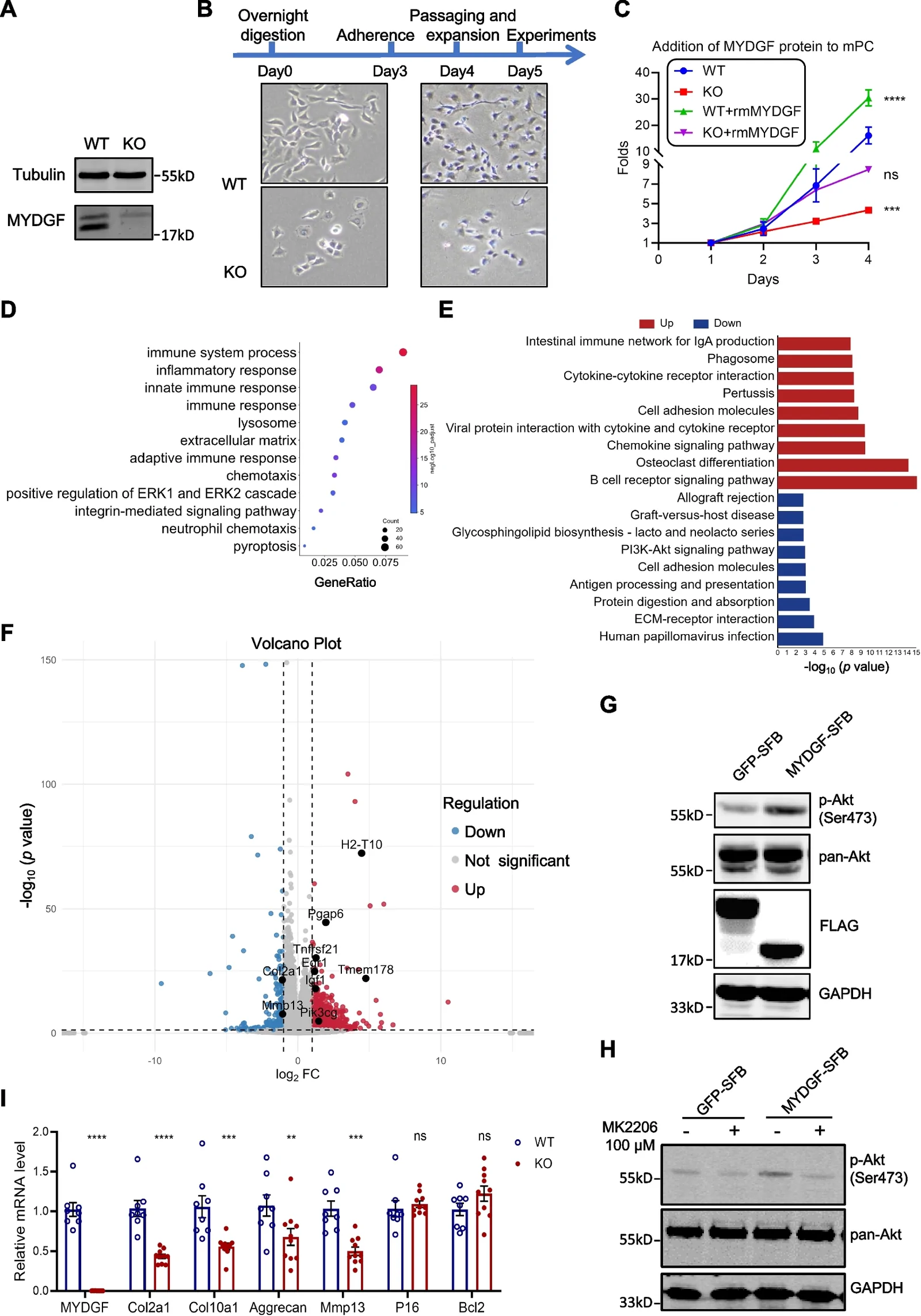
MYDGF deficiency impairs chondrocyte proliferation and alters cartilage-related gene expression profiles. **A** Western blot analysis confirming MYDGF expression in WT and MYDGF KO cells. Tubulin was used as a loading control. **B** Experimental timeline for isolation, plating, passaging, and downstream analyses of mouse primary chondrocytes (mPCs). *n* = 3 each group. **C** Growth curves of WT and KO mPCs treated with recombinant MYDGF protein. Cell proliferation was monitored over four days, showing partial rescue of KO cell growth by exogenous MYDGF. Statistical significance was determined by one-way ANOVA with Dunnett's multiple comparisons test versus WT. *n* = 3 each group. **D** Gene Ontology (GO) enrichment analysis showing overrepresentation of immune and inflammatory response–associated biological processes in MYDGF-deficient mPCs. **E** KEGG pathway enrichment analysis of differentially expressed genes in KO versus WT mPCs, highlighting pathways related to cytokine signaling, extracellular matrix (ECM)-receptor interaction, and PI3K–Akt signaling et al. **F** Volcano plot illustrating differentially expressed genes between WT and KO mPCs identified by RNA sequencing. Upregulated and downregulated genes are indicated. **G** Western blot analysis showing increased Akt phosphorylation at Ser473 in HeLa cells overexpressing MYDGF-SFB compared with GFP-SFB control. **H** Western blot analysis showing that pharmacological inhibition of Akt signaling with MK2206 (100 μM) suppresses MYDGF-induced Akt phosphorylation. **I** Quantitative PCR analysis of chondrogenic, extracellular matrix, senescence- and survival-related genes in WT and KO mPCs, including MYDGF, Col2a1, Col10a1, Aggrecan, Mmp13, P16, and Bcl2, normalized to mTubulin. Statistical significance was determined by two-tailed unpaired Student's *t*-test. *n* = 9 biological replicates (mice). All data were presented as mean ± SD. Statistical significance was set at *p < 0.05, **p < 0.01, ***p < 0.001 and ****p < 0.0001

To further investigate the mechanism, primary chondrocytes were isolated from WT and KO mouse. Primary chondrocytes from MYDGF KO mice showed no detectable MYDGF protein by western blot, in contrast to robust expression in WT cells (Fig. 6A). KO chondrocytes also exhibited an altered morphology, appearing more flattened and spread out compared to the compact, rounded shape of WT controls (Fig. 6B). Functionally, MYDGF ablation severely impaired chondrocyte proliferation. CCK-8 assays demonstrated a significantly reduced growth rate in KO cells, which was restored to a level not significantly different from WT cells upon supplementation with purified recombinant MYDGF protein (Fig. 6C). This confirmed that the proliferative defect was directly attributable to MYDGF loss.

To uncover the underlying transcriptional alterations, we performed RNA sequencing. MYDGF KO chondrocytes displayed widespread transcriptomic reprogramming (*n* = 9, each group). Gene Ontology enrichment highlighted a pronounced shift toward immune and inflammatory activation, including processes such as immune system process, inflammatory response, and both innate and adaptive immune response (Fig. 6D). Increases in chemotaxis and pyroptosis were also noted.

KEGG pathway analysis further revealed suppression of key anabolic and pro-survival pathways, most notably the PI3K–Akt signaling pathway, which was significantly downregulated (Fig. 6E). Concurrently, among pathways associated with matrix degradation and immune activation, ECM-receptor interaction was downregulated, whereas osteoclast differentiation and chemokine signaling pathways were activated. In the volcano plot of differentially expressed genes, we observed not only significant alterations in genes associated with the PI3K–Akt signaling pathway, such as Igf1 and Prkcz, but also notable changes in the expression of matrix-related genes, including Col2a1 and Mmp13, which are involved in extracellular matrix synthesis and degradation (Fig. 6F).

Given the central role of PI3K-Akt in chondrocyte survival and matrix homeostasis, we tested whether MYDGF is involved in regulation of this pathway. Overexpression of MYDGF markedly increased Akt phosphorylation at Ser473 (Fig. 6G), and this enhanced activation was blocked by the Akt inhibitor MK2206 (Fig. 6H), suggesting that MYDGF is associated with PI3K-Akt signaling pathway, though the precise relationship remains to be defined. RT–qPCR analysis validated the RNA-seq results, showing coordinated changes in cartilage-related gene expression, including downregulation of Col2a1, Aggrecan, Col10a1, and Mmp13 in MYDGF-deficient cells (Fig. 6I), indicative of disrupted matrix homeostasis.

In summary, loss of MYDGF is associated with disrupted chondrocyte proliferation and correlates with a broad transcriptomic shift suggestive of a transition from an anabolic, matrix-stabilizing state toward a catabolic and inflammatory phenotype. This transcriptional reprogramming is characterized by suppressed PI3K–Akt signaling and activation of immune-related pathways, offering a potential molecular basis for the functional decline of MYDGF-deficient chondrocytes and their increased susceptibility to degeneration. Collectively, these cellular insights provide a framework for understanding how MYDGF deficiency may precipitate tissue-level pathology in osteoarthritis.

## Conclusion

This study identifies, through a genome-wide CRISPR-Cas9 screen, the myeloid-derived growth factor (MYDGF) as a novel positive regulator of telomerase activity and delineates its critical role in maintaining cartilage homeostasis and mitigating osteoarthritis (OA) progression. Our findings reveal that MYDGF preserves chondrocyte function through multiple mechanisms: it directly enhances telomerase activity via an endocytosis-dependent, calcium-mediated signaling pathway, while also being associated with activation of the pivotal PI3K–Akt pro-survival and proliferation cascade, and these two signaling events may operate in parallel or partially independently. The synergistic action of these pathways underpins the chondrocyte's anabolic capacity, stress resilience, and resistance to premature functional decline. Conversely, MYDGF deficiency initiates a cascading vicious cycle culminating in the structural disintegration of cartilage, offering a fresh perspective on OA pathogenesis.

We first establish MYDGF as an essential upstream factor required for maintaining basal telomerase activity. In HeLa cells, loss or gain of MYDGF function directly and reversibly modulates TERT transcription and telomerase activity. Significantly, exogenous recombinant MYDGF protein stimulates telomerase in a dose-dependent manner across diverse cell types, a process dependent on clathrin-mediated endocytosis and intra- and extracellular calcium signaling. This suggests MYDGF may act as a diffusible paracrine signal, converting an extracellular cue into an intracellular event regulating telomerase via specific receptor-mediated internalization. For long-lived chondrocytes constantly exposed to mechanical and inflammatory stress, telomerase activity likely contributes to cellular protection through both canonical telomere maintenance and non-canonical functions, including anti-apoptotic signaling and stress responses. Therefore, by sustaining telomerase function, MYDGF provides crucial "molecular buffering" capacity, enabling chondrocytes to cope with persistent micro-damage within the joint, although the contribution of non-canonical pathways warrants further investigation.

Furthermore, we demonstrate that MYDGF functions as a key upstream regulator of chondrocyte homeostasis by coordinating intracellular survival signaling with extracellular matrix (ECM) dynamics. Loss of MYDGF leads to a marked reduction in Akt phosphorylation, accompanied by broad transcriptional reprogramming characterized by downregulation of anabolic genes such as Col2a1 and Aggrecan, along with alterations in catabolic and inflammatory pathways. Together with the concurrent decline in telomerase activity, these changes likely constrain chondrocyte proliferation, impair functional stability, and predispose cells to premature aging or dedifferentiation. Importantly, these intracellular defects are further propagated to the level of matrix regulation. Rather than driving a classical catabolic shift, MYDGF deficiency appears to induce a state of globally reduced matrix turnover, as evidenced by the simultaneous downregulation of structural components (Col2a1, Aggrecan) and remodeling-associated factors (Col10a1 and Mmp13). Given that cartilage homeostasis depends on a dynamic equilibrium between matrix synthesis and degradation, the coordinated but imbalanced downregulation of ECM-related genes in MYDGF-deficient chondrocytes results in an imbalanced ECM state characterized by predominant downregulation of matrix synthesis relative to degradation. Under this condition, matrix deposition (e.g., Col2a1, Aggrecan) is disproportionately compromised, while residual catabolic activity—including other MMPs and ADAMTSs that are not significantly downregulated—may still contribute to matrix degradation. Consequently, the overall amount of cartilage matrix decreases, leading to accelerated cartilage degeneration despite reduced Mmp13 transcription. Collectively, these findings suggest that MYDGF is not only required for maintaining pro-survival signaling and proliferative capacity but also plays an essential role in preserving the dynamic remodeling potential of cartilage, thereby enabling effective adaptation to mechanical stress and injury. At the tissue and animal model level, these cellular defects translate directly into severe OA pathology. Consistent with our findings in mice, analysis of a public human dataset (GSE55235) showed increased MYDGF expression in arthritic synovium relative to normal tissues ((Extended Data Fig. 4), further supporting the clinical relevance of MYDGF dysregulation in OA. In the DMM-induced OA model, MYDGF KO mice exhibit accelerated cartilage degradation, proteoglycan loss, osteophyte formation, and aberrant subchondral bone remodeling. These phenotypes reflect the loss of core assembly functions and the impairment of stress responses, which leads to the failure of matrix maintenance and repair functions. As a result, the vulnerable cartilage in the body degrades rapidly under mechanical stress. Importantly, our rescue experiments demonstrate that local intra-articular delivery of MYDGF via an AAV vector effectively attenuates cartilage destruction, preserves matrix components, and improves bone abnormalities. This suggests that MYDGF loss results in OA progression, and its restoration can rebuild protective signaling within the joint. Nevertheless, we acknowledge that direct evidence of transduction efficiency is lacking, and future studies incorporating such assessments will be essential to fully validate and optimize AAV‑mediated MYDGF delivery for clinical translation.

The protective role of MYDGF may be particularly relevant within the unique microenvironment of the OA joint. Chronic hypoxia is a common feature, and hypoxia-inducible factor HIF1α has been reported to upregulate MYDGF expression. Thus, under physiological or early pathological conditions, the hypoxic joint microenvironment may activate an endogenous protective program via the HIF1α-MYDGF axis to enhance chondrocyte survival and adaptation to stress. Loss of MYDGF severs this important adaptive signal, rendering chondrocytes more vulnerable to oxidative stress, mechanical loading, and inflammatory insult. Moreover, dysfunctional chondrocytes may exacerbate local tissue damage by secreting senescence-associated secretory phenotype (SASP) or other inflammatory factors, triggering secondary inflammation and creating a vicious cycle that ultimately drives the severe OA phenotype.

**Fig. 7:**
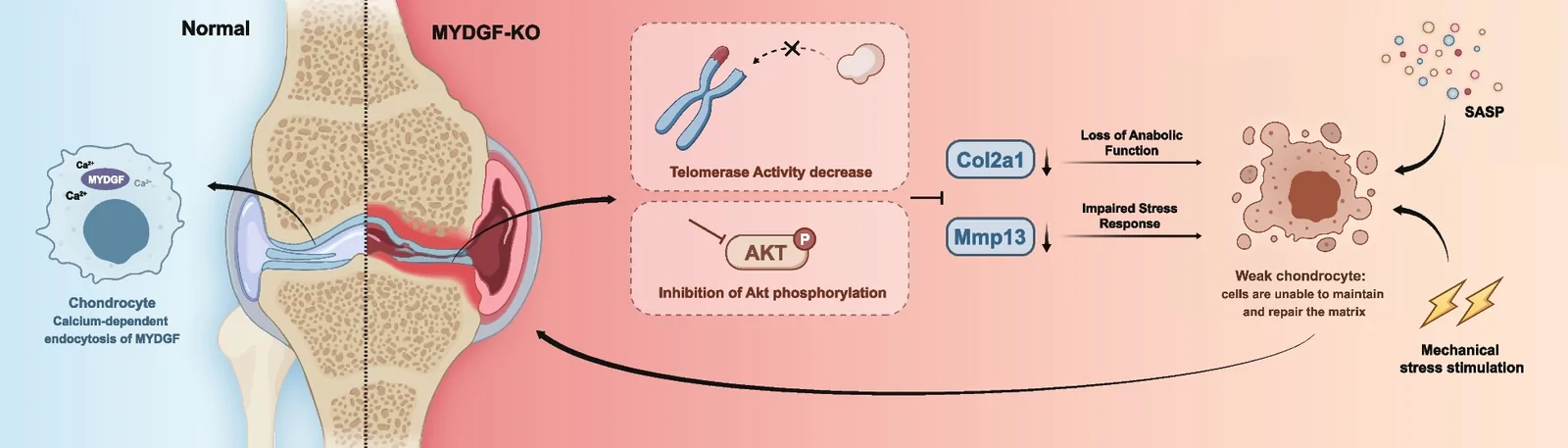
Mechanism diagram of MYDGF. In normal chondrocytes, MYDGF uptake is Ca^2^⁺-dependent, which sustains telomerase activity and Akt phosphorylation. Loss of MYDGF leads to decreased telomerase activity and impaired Akt phosphorylation, resulting in reduced anabolic capacity (e.g., Col2a1) and suppressed matrix remodeling (Mmp13). These changes weaken chondrocyte function in maintaining cartilage homeostasis. Under mechanical stress, dysfunctional chondrocytes exhibit impaired stress responses and increased SASP, ultimately contributing to cartilage degeneration

A schematic model summarizing our proposed mechanism is presented in Fig. 7. Rather than representing isolated molecular events, our data support a coordinated failure of chondrocyte homeostasis upon MYDGF loss, linking impaired intracellular signaling to defective extracellular matrix regulation. In this framework, disruption of pro-survival and stress-adaptive pathways converges on a state of compromised matrix turnover, where the balance between matrix production and renewal is broadly suppressed. As a consequence, chondrocytes progressively lose their capacity to maintain tissue integrity under mechanical and inflammatory stress, ultimately adopting a dysfunctional phenotype associated with senescence-like features and altered secretory activity. This integrated model provides a conceptual basis for understanding how MYDGF deficiency translates from molecular perturbations to cartilage degeneration and OA progression.

In summary, our work positions MYDGF as a central player in cartilage health, where it cooperatively regulates telomerase activity and Akt survival signaling to maintain chondrocyte anabolic function and stress resilience. MYDGF deficiency initiates a domino effect from cellular energy crisis and functional decline to tissue breakdown. These findings deepen the understanding of OA molecular mechanisms and highlight MYDGF, along with its downstream pathways, as promising therapeutic targets. Our current data support an effect of MYDGF on telomerase activity; however, whether MYDGF can induce telomere elongation in vivo remains unknown. Therefore, telomerase activation should be considered a potentially important contributor to the protective effects of MYDGF in OA, pending definitive causal validation.

Furthermore, there are still several limitations in terms of the mechanisms. First, the receptor through which MYDGF signals in chondrocytes remains unidentified, leaving open how extracellular MYDGF is converted into intracellular calcium fluxes and Akt phosphorylation. Second, while our data indicate that the endocytosis-dependent calcium pathway and the PI3K-Akt cascade may operate in parallel or partially independently, the temporal and causal relationship between these two branches has not been established. To gain initial insight into downstream effectors, we performed preliminary experiments in HeLa cells. As shown in Extended Data Fig. 5A–D, MYDGF stimulation upregulated the expression of c-Myc and SP1, both of which are known Akt downstream targets and transcriptional regulators of TERT. Furthermore, shRNA-mediated knockdown of c-Myc (Extended Data Fig. 5E) markedly reduced telomerase activity and abrogated the responsiveness to exogenous MYDGF stimulation (Extended Data Fig. 5 F), indicating that c-Myc is required for MYDGF-induced telomerase activation in this context. However, these data were obtained in a heterologous cell line and require systematic validation in primary chondrocytes and in vivo. Therefore, the proposed "multi-mechanism" model should be viewed as a framework of parallel contributory pathways rather than a fully defined hierarchical network.

Future work should aim to identify the specific receptor(s) for MYDGF in joint cells, fully elucidate its downstream signaling network, explore whether forced telomerase activation can rescue the OA phenotype in the absence of MYDGF, and assess the translational potential of MYDGF-based therapies. In particular, long-term treatment models in humanized mice combined with direct telomere length measurements will be necessary to determine whether MYDGF induces telomere elongation in vivo.

## Supplementary Information


Supplementary Material 1.

## Data Availability

The datasets generated and/or analyzed during the current study are available from the corresponding author upon reasonable request. The RNA-seq raw data has been submitted to the SRA database under accession number PRJNA1510871.
